# Botulinum Toxin A for Hair Loss Treatment: A Systematic Review of Efficacy, Safety, and Future Directions

**DOI:** 10.1016/j.jpra.2023.09.006

**Published:** 2023-09-21

**Authors:** Ramadan S. Hussein, Salman Bin Dayel, Othman Abahussein

**Affiliations:** Dermatology Unit, Department of Internal Medicine, College of Medicine, Prince Sattam Bin Abdulaziz University, Al-Kharj, Saudi Arabia

**Keywords:** hair loss, botulinum toxin, management

## Abstract

**Background:**

Hair loss is a common condition with significant impact globally, yet its treatment efficacy and safety remain debated. Botulinum toxin A (BoNT-A) has emerged as a potential therapeutic option, but a comprehensive review on this topic is lacking**.**

**Objective:**

This review critically evaluates the current evidence on BoNT-A for hair loss treatment, highlighting the gaps in previous reviews and providing a comprehensive analysis of its efficacy, safety, and future prospects.

**Methods:**

A systematic search of electronic databases identified relevant studies published up to September 2022**.**

**Results:**

Prior reviews primarily focused on androgenetic alopecia and lacked the evaluation of other alopecia types and underlying mechanisms. Our review addresses this gap, incorporating a broader spectrum of hair loss conditions. Mechanisms of BoNT-A in hair growth modulation, potential side effects, and future research directions are discussed.

**Conclusion:**

This review adds to the existing body of knowledge by providing a comprehensive evaluation of BoNT-A in hair loss treatment. The findings will serve as a foundation for further research and guide clinicians in making informed decisions, ultimately improving the outcomes and quality of life for individuals suffering from hair loss.

## Introduction

Hair loss, a condition that transcends generations and cultures, has been a persistent concern affecting millions worldwide. The profound impact of hair loss on individual well-being cannot be understated; it evokes psychological and emotional distress that resonates beyond the realm of physical appearance. While the prevalence of hair loss is ubiquitous, effective treatment options that deliver consistent and satisfactory outcomes have remained elusive.

Diverse factors contribute to hair loss, ranging from genetic predispositions and hormonal imbalances to medical conditions, nutritional deficiencies, and lifestyle influences.^1^ Despite its ubiquity, the armamentarium of available treatments has been characterized by limitations and variability in efficacy. The current array of therapeutic approaches encompasses topical and oral medications, hair transplant surgery, and laser therapy.^2^ However, these methodologies present challenges. Topical and oral medications, such as minoxidil and finasteride, necessitate long-term use and may yield variable results. Hair transplant surgery, though effective, is minimally invasive, and has dynamic nature of costs, but not universally suitable. Laser therapy, while noninvasive, exhibits mixed efficacy and requires prolonged treatment sessions.^3^ The persistent gaps in treatment options underscore the need for innovative interventions that can revolutionize the field of hair loss management.

In recent years, the potential of botulinum toxin type A (BoNT-A) as an unconventional therapeutic modality for hair loss has ignited interest. BoNT-A, renowned for its aesthetic and medical applications, has emerged as a promising candidate in the realm of hair restoration.^4^^,^^5^ Its innovative mechanism of action, characterized by neuromuscular blockade and modulation of cellular pathways, presents an alternative avenue for addressing hair loss and promoting regrowth. However, while preliminary studies suggest potential benefits, a comprehensive evaluation of the available evidence on BoNT-A's efficacy, safety, and future prospects in the context of hair loss is essential.

This comprehensive review seeks to critically assess the current body of knowledge surrounding the use of BoNT-A in hair loss treatment. We aim to bridge the existing gaps in the literature by encompassing a broader spectrum of hair loss conditions beyond the confines of androgenetic alopecia (AGA), the most studied type. By delving into the mechanisms underlying BoNT-A's role in hair growth modulation, potential side effects, and avenues for future research, we intend to present a well-rounded analysis that serves as a foundational resource for both clinicians and researchers.

While previous reviews have provided valuable insights, they have often focused on specific aspects of hair loss, lacked consistent reporting on key factors, and neglected to explore the full breadth of underlying mechanisms.^6^ By addressing these limitations, we endeavor to furnish readers with a holistic perspective that not only evaluates BoNT-A's efficacy in various hair loss conditions but also outlines the path ahead for refining its clinical applications. The information contained within this review is poised to guide healthcare professionals in making informed decisions, thus contributing to improved patient outcomes and enhancing the quality of life for those grappling with the distressing impact of hair loss.

## Objective and Methods

The main aim of this review is to critically assess the existing evidence concerning the utilization of BoNT-A in treating hair loss. Our goal is to address the research gap by conducting a thorough analysis of BoNT-A's effectiveness, safety, and potential for future research.

To accomplish this objective, we conducted a systematic and comprehensive literature search, aligned with the Preferred Reporting Items for Systematic Reviews and Meta-Analyses (PRISMA) guidelines**.** Prior to conducting the systematic review, we developed a detailed protocol outlining the research objectives, inclusion and exclusion criteria, search strategy, data extraction methods, and assessment of study quality. Search was conducted across electronic databases such as PubMed, Embase, and Cochrane Library. Relevant studies published until September 2022, written in English language, regardless of their study design, to ensure a comprehensive examination of the available evidence, were included. The search strategy incorporated a combination of keywords related to hair loss, alopecia, BoNT-A, and treatment. We employed rigorous criteria for study selection based on relevance to BoNT-A's use in hair loss treatment. Two independent reviewers conducted the study selection process, and any discrepancies were resolved through consensus. Data from the selected studies were systematically extracted, and methodological quality and level of evidence were critically assessed using established tools such as the Oxford Center and Jadad scores. The findings from the selected studies were synthesized and presented in a structured manner, addressing various aspects of BoNT-A therapy for hair loss treatment. This synthesis provides a comprehensive overview of the available evidence.

## Previous Published Reviews

### Lacuna in the Existing Literature

The existing literature on the use of BoNT-A in the treatment of hair loss has some notable gaps. Previous reviews have primarily focused on limited aspects, such as AGA, while neglecting a comprehensive evaluation of other types of alopecia, including alopecia areata, traction alopecia, and telogen effluvium. This lack of inclusion limits the overall understanding of BoNT-A's potential in addressing a broader spectrum of hair loss conditions. In addition, previous reviews have not adequately explored the underlying mechanisms of action of BoNT-A in hair growth modulation.

### Limitations of Previous Reviews

Several limitations can be identified in the previous reviews on the use of BoNT-A in hair loss treatment. Firstly, there is often a lack of standardized inclusion criteria and study selection methods, which may introduce selection bias and limit the generalizability of the findings. Some reviews have also relied heavily on a single study design or failed to provide a comprehensive synthesis of the available evidence. In addition, the limited sample sizes and short follow-up durations in some studies included in previous reviews may affect the robustness of the conclusions. Furthermore, there has been a lack of consistent reporting on important factors such as treatment protocols, outcomes, and adverse effects. These limitations highlight the need for a more rigorous and comprehensive review that addresses these gaps and provides a more reliable analysis of BoNT-A's efficacy, safety, and potential mechanisms of action in the treatment of hair loss.^6^

## Efficacy of BoNT-A in Hair Loss Treatment

### Evaluation of Studies on AGA

The most prevalent type of hair loss is AGA, which is commonly referred to as male or female pattern baldness. Numerous studies have investigated the efficacy of BoNT-A in the treatment of AGA. This section of the review evaluates the methodology, treatment protocols, outcome measures, and results of these studies.

The evaluation of studies on AGA includes assessing parameters such as hair regrowth, hair density, hair diameter, and patient satisfaction. The review examines the various types of study designs, such as observational studies and randomized controlled trials, to determine the level of evidence and the overall efficacy of BoNT-A in promoting hair growth and reducing hair loss in individuals with AGA (Table 1).^6^

**Table 1:** Summary of clinical studies investigating the use of botulinum toxin A (BoNT-A) for hair loss treatment.^7^, ^8^, ^9^, ^10^, ^11^

Author	Study	Author	Type of Alopecia	Methodology	Treatment Protocol	Outcome Measures	Results	Side Effects	Level of Evidence
Freund et al. 2010	Study 1	Freund et al. 2010	Androgenetic Alopecia	Pilot Study	Intramuscular BoNT-A injections (150 units) into specified muscles	Hair density increase within a 2-cm area, overall response rate	18% increase in hair density, 75% overall response rate	No adverse events reported	Low
Singh et al. 2017	Study 2	Singh et al. 2017	Androgenetic Alopecia	Prospective Cohort Study	Intramuscular BoNT-A injections (150 units) into specified muscles	Photographic and self-assessment of response	80% excellent photographic response, 70% good-to-excellent self-assessment response	No adverse events reported	Low
Zhang et al. 2019	Study 3	Zhang et al. 2019	Androgenetic Alopecia	Prospective Cohort Study	Intramuscular BoNT-A injections (50 units) into multiple sites	Hair count changes, response rate	44% had >10% hair count changes, 79.1% response rate	No adverse events reported	Low
Shon et al. 2020	Study 4	Shon et al. 2020	Androgenetic Alopecia	Prospective Cohort Study	Intradermal BoNT-A injections (30 units) into specified sites	Hair per square centimeter change	5.1% increase in hair per square centimeter	No adverse events reported	Low
Zhou et al. 2020	Study 5	Zhou et al. 2020	Androgenetic Alopecia	Randomized Trial	Intramuscular BoNT-A injections (100 units) into specified muscles in sessions	Hair counts change	Significant increase in hair counts in both groups	Transient side effects reported by five subjects	High

*Note:* The level of evidence is classified as "Low" due to limitations in study design and sample size

### Assessment of Studies on Cephalalgia Alopecia, Alopecia Areata, and Folliculiti Decalvans

In addition to AGA, other forms of hair loss, such as alopecia areata, traction alopecia, and telogen effluvium, also have a significant impact on individuals. This section of the review expands the analysis to include studies that have investigated the use of BoNT-A in these specific types of alopecia (Table 2).

**Table 2:** Using botulinum toxin in treating alopecia, other than AGA.^13^^,^^14^^,^^5^^,^^15^

Study	Author	Type of Alopecia	Methodology	Treatment Protocol	Outcome	Result	Side Effects	Level of Evidence
Study 1	FM Cutrer and colleagues, 2006:	Alopecia areata (Cephalalgic)	Retrospective, Case Report	Not mentioned; A total of 492.5 units in three times of injection	Good response	The patient was satisfied with the result	No AEs reported	Low
Study 2	Tamura BM and colleagues, 2007:	Folliculitis decalvans	Retrospective, Case Report	100 units reconstituted with 4 ml saline solution, dosing of 60, 80, 100, and 150 units for four different individuals	Two patients controlled the secretion and had hair growth of alopecic areas, one had reduced secretion and partial hair growth, and one had little response	Response rate: 79%	No Adverse Events (AEs) reported	Low
Study 3	Hee-Ryung Cho and colleagues, 2010:	Alopecia areata	Prospective, Pilot Study	Not mentioned; A total of 60 units	only one of seven patients have spontaneously hair growth of positive outcomes	N/A	No AEs reported	Low
Study 4	Pablo Irimia and colleagues, 2013	Cephalalgic alopecia	Retrospective, Case Report	100 units per injection	Onabotulinum toxin A was injected into frontalis, temporalis trapezius, and occipitalis muscles around the site of the pain (100 units)	Excellent response	No AEs reported	Low

The assessment of studies on alopecia areata focuses on evaluating the efficacy of BoNT-A in inducing hair regrowth and reducing the extent of hair loss in individuals with this autoimmune condition. Studies on Cephalalgia alopecia and folliculitis are also analyzed to determine the potential benefits of BoNT-A in these conditions.^12^

## Mechanisms of Action

### Understanding the Role of BoNT-A in Hair Growth Modulation

BoNT-A primarily acts by inhibiting the release of neurotransmitters, particularly acetylcholine, at the neuromuscular junction. However, its role in hair growth modulation extends beyond its neuromuscular effects.^16^ BoNT-A is believed to influence the hair follicle cycle and its associated cellular processes.

**Figure 1:**
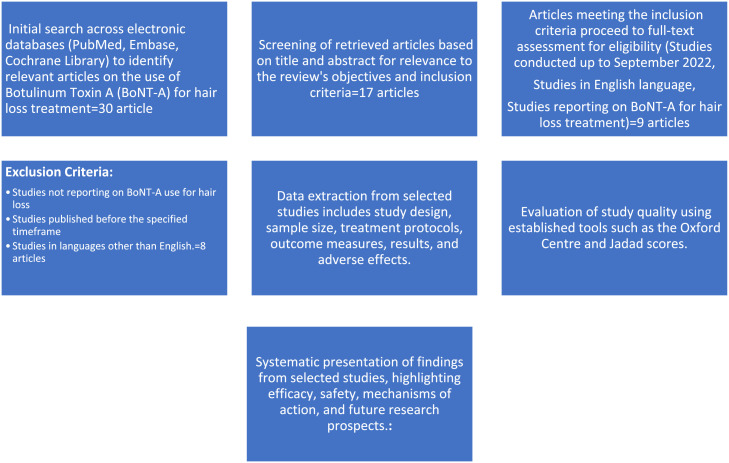
Systematic Search and Study Selection Process flowchart

The hair follicle undergoes a cyclic process comprising three stages: anagen (growth phase), catagen (transition phase), and telogen (resting phase). BoNT-A is thought to prolong the anagen phase by reducing the activity of hair follicle dermal papilla cells (DPCs), which affects the hair growth rate and duration. In addition, BoNT-A may inhibit the production of proinflammatory cytokines and modulate the interaction between hair follicle cells and immune cells, potentially contributing to its effects on hair growth.^11^ Botulinum toxin injection has the ability to induce muscle relaxation, alleviate pressure on blood vessels, and enhance blood circulation, leading to increased transcutaneous oxygen levels. This improved blood flow can also contribute to the elimination of accumulated dihydrotestosterone (DHT), thereby reducing the signal for hair follicle miniaturization (Figure 1).^8^

### Potential Pathways Involved

Several potential pathways have been proposed to elucidate the mechanisms by which BoNT-A influences hair growth modulation. These pathways include the following:

Neural Pathway: BoNT-A's primary mechanism of action involves blocking the release of acetylcholine, a neurotransmitter that plays a role in the communication between nerves and muscles. By inhibiting acetylcholine release at the neuromuscular junctions associated with hair follicles, BoNT-A may indirectly affect hair growth.^16^

Inflammatory and Immune Pathways: Studies have demonstrated that BoNT-A can regulate the generation and release of proinflammatory cytokines, such as tumor necrosis factor-alpha and interleukins. By reducing inflammation in the scalp, BoNT-A may create a more favorable environment for hair growth. Moreover, BoNT-A's effects on immune cells and their interaction with hair follicle cells may play a role in regulating hair growth.^17^

Growth Factor Pathways: BoNT-A has been observed to interact with different growth factors essential for the development and upkeep of hair follicles, including fibroblast growth factor, insulin-like growth factor, and vascular endothelial growth factor. These interactions have the potential to affect the growth and specialization of hair follicle cells, ultimately influencing hair growth.^18^ Moreover, DHT stimulates the production of transforming growth factor-β1 (TGF-B1) in DPCs, which in turn inhibits the growth of follicular epithelial cells. Therefore, TGF-β1 plays a significant role in AGA, and blocking its effects could potentially prevent AGA.^19^ Botulinum toxin type A (BoNT) has shown the ability to suppress the secretion of TGF-β1 from DPCs. When BoNT is injected into the skin, it effectively combats AGA by inhibiting TGF-β1 secretion in the hair bulb, which is known to impede the growth of follicular keratinocytes and disrupt the hair cycle.^20^

## Safety Profile and Adverse Effects

Overall, BoNT-A is considered to have a favorable safety profile when used appropriately by trained healthcare professionals. However, like any medical intervention, there are potential risks and adverse effects that need to be considered. It is crucial to weigh the potential benefits against the risks before initiating BoNT-A treatment for hair loss.^6^

Common Side Effects and Their Management:

*Injection Site Reactions*: The most frequent negative effects of BoNT-A treatment are reactions at the injection site. These include redness, swelling, bruising, and pain at the injection site. These reactions are typically transient and resolve spontaneously within a few days to weeks. The use of ice packs, topical anesthetics, or analgesics can help alleviate discomfort.^11^

*Temporary Muscle Weakness*: BoNT-A's mechanism of action involves temporary muscle paralysis, which can lead to muscle weakness in the treated area. This effect is usually localized and resolves over time. It is important to inform patients about this potential side effect to manage their expectations and minimize any functional limitations.^21^

*Systemic Effects*: Although rare, BoNT-A can have systemic effects when administered at higher doses or when it spreads beyond the intended treatment area. Systemic effects may include generalized muscle weakness, swallowing difficulties, or respiratory compromise. Adherence to proper injection techniques and dosing guidelines can minimize the risk of systemic effects.^15^

It is crucial for healthcare providers to be knowledgeable about the potential side effects of BoNT-A and to inform patients about them. Proper patient selection, individualized treatment planning, and adherence to recommended dosages and injection techniques are essential for ensuring safety during BoNT-A administration.^11^

Close monitoring of patients during the treatment period is important to identify any adverse effects promptly. Patient education on recognizing and reporting any concerning symptoms is also essential. In the event of any severe or persistent adverse effects, appropriate medical intervention and follow-up should be provided.^6^

## Future Research Directions

Table 3

**Table 3:** Organized presentation of the future research directions, areas for investigation, and potential advancements in the use of BoNT-A for hair loss treatment.^6^

Future Research Directions	Areas for Further Investigation	Novel Approaches and Potential Advancements
Optimal Treatment Protocols	- Determine the most effective dosage, frequency, and duration of BoNT-A treatment for different types of hair loss.	- Explore combination therapies with other hair growth-promoting agents.
Long-Term Follow-up	- Conduct longitudinal studies with extended follow-up periods to assess the durability of treatment outcomes and the need for maintenance treatments.	- Investigate targeted delivery systems or formulations to enhance BoNT-A's local effects.
Comparative Studies	- Compare the efficacy, safety, and patient satisfaction of BoNT-A with existing treatment modalities for hair loss.	- Utilize advanced imaging techniques (e.g., dermoscopy, Optical Coherence Tomography) to assess hair follicle changes following BoNT-A treatment.
Mechanistic Studies	- Explore the underlying molecular pathways and cellular processes involved in BoNT-A's hair growth modulation.	- Investigate personalized medicine approaches based on individual patient factors that influence treatment response.
Potential Advancements		
Combination Therapies	- Investigate the synergistic effects of combining BoNT-A with other hair growth-promoting agents, such as minoxidil or PRP.	
Targeted Delivery Systems	- Develop innovative delivery methods (e.g., microneedle arrays, liposomal formulations) to enhance the penetration and bioavailability of BoNT-A within the hair follicles.	
Personalized Medicine	- Investigate individual patient factors (genetic variations, hormonal status, and immune profiles) that influence the response to BoNT-A treatment and tailor protocols accordingly.	
Advanced Imaging Techniques	- Utilize high-resolution dermoscopy or Optical Coherence Tomography to provide detailed assessments of hair follicle morphology and density changes following BoNT-A treatment.	

### Limitations

Only articles written in English and containing full texts were included. If studies included both adult and pediatric data, they were only considered if they provided individual-level data specifically for pediatric patients. No meta-analysis was conducted.

## Conclusion

Efficacy of BoNT-A: BoNT-A has shown promising results in promoting hair growth and reducing hair loss in various types of hair loss conditions. BoNT-A can be considered as a potential therapeutic option for individuals with different types of hair loss. Healthcare professionals should be knowledgeable about the optimal treatment protocols, potential side effects, and management strategies. Patient selection, individualized treatment planning, and proper follow-up are crucial for achieving favorable outcomes. However, further research is needed to optimize eatment protocols and determine its long-term effects.
